# The Illusion of Disapproval: Pluralistic Ignorance and Attitudes Toward Tattooed Teachers

**DOI:** 10.3390/ejihpe16080115

**Published:** 2026-08-11

**Authors:** Dániel Kormos, Attila Lengyel, Anetta Müller, Éva Bácsné Bába, Antonia Kinczel, Melinda Bíró

**Affiliations:** 1 Institute of Sport Science Coordination, University of Debrecen, 4032 Debrecen, Hungary; kddaniel099@gmail.com (D.K.); biro.melinda@sport.unideb.hu (M.B.); 2 Coordination and Research Centre for Social Sciences, Faculty of Economics and Business, University of Debrecen, 4032 Debrecen, Hungary; lengyel.attila@econ.unideb.hu; 3 Institute of Sports Economics and Management, Faculty of Economic Sciences, University of Debrecen, 4032 Debrecen, Hungary; muller.anetta@econ.unideb.hu (A.M.); bacsne.baba.eva@econ.unideb.hu (É.B.B.); 4 Institute of Sports Economics and Management, Faculty of Economic Sciences, Doctoral School of Management and Business, University of Debrecen, 4032 Debrecen, Hungary

**Keywords:** attitudes toward tattooed teachers, body art, educational groups, pluralistic ignorance, professional socialization, teacher appearance norms

## Abstract

Background: Tattooed teachers are increasingly visible in schools, yet the norms governing their professional acceptability remain poorly understood. This study tests whether pluralistic ignorance—private acceptance systematically exceeding the acceptance attributed to others—operates in attitudes toward tattooed teachers, and identifies individual-level predictors of this misperception. Methods: A cross-sectional online survey was conducted with 1226 individuals in Hungary across four groups: physical education (PE) teacher trainees, university students from other disciplines, secondary school students, and in-service teachers. Participants rated their own acceptance of tattooed PE and academic-subject teachers and the acceptance they attributed to other teachers and to parents, and reported tattoo status, gender, and professional background. Results: Own acceptance exceeded attributed acceptance in all four groups and for both reference targets, consistent with pluralistic ignorance; gaps were largest among secondary school students and smallest—though still positive—among in-service teachers. Personal tattoo experience was the strongest individual predictor of acceptance after group membership, and among teachers both age and experience were negatively associated with acceptance. Acceptance was predominantly conditional, structured by a visibility hierarchy in which arm placements were most acceptable and facial or neck placements least so. Conclusions: The apparent disapproval of tattooed teachers reflects collective misperception more than genuine consensus, suggesting that making actual peer attitudes visible could reduce conformity pressure and support more authentic professional self-expression.

## 1. Introduction

People routinely and systematically misjudge what others think. When individuals misperceive the attitudes of their colleagues, students, or parents, they may conform to a norm that rests on no true consensus—suppressing authentic expression in response to a disapproval that exists largely in their own minds. The result is a self-sustaining false norm that gains traction not through shared belief but through shared silence. The present research examines whether this dynamic operates in the domain of professional appearance norms in education, focusing on attitudes toward tattooed teachers across four educational groups in Hungary: physical education (PE) teacher trainees, university students from other disciplines, secondary school students, and in-service teachers.

A central distinction separates descriptive norms—what people actually think or do—from injunctive norms—what people believe others approve or disapprove of. When direct access to others’ private attitudes is limited, individuals infer the normative climate from public behaviour and cultural cues, a process prone to systematic error. Such distortions are well documented in educational settings: teachers form expectations of students that show considerable stability over time (Hao et al., 2022; Lomholt, 2026), perceptions of what is normal predict intergroup attitudes and wellbeing (Ward et al., 2025), and the anxiety of being socially excluded on the basis of assumed—often mistaken—group views can drive conformity and self-censorship (Wang et al., 2025; Konieczna, 2024). Appearance norms in particular operate as powerful institutional constraints even when they are not, in fact, widely endorsed (Ederoclite et al., 2025).

Tattooing is not merely an individual esthetic choice but a cultural practice whose social meaning has shifted profoundly within living memory. For much of the twentieth century, Western sociological and clinical writing framed body art through the lens of deviance, associating it with marginal, criminal, or subcultural populations (Lane, 2014). Since the 1990s, however, tattooing has undergone what has been characterized as an artification: a movement from stigmatized craft to a recognized cultural field with its own esthetic conventions, professional standards, and claims to artistic legitimacy (Kosut, 2014). In parallel, the practice was absorbed into mainstream consumer culture, popularized through media visibility and celebrity adoption, and increasingly consumed as a vehicle of personal self-expression rather than of group affiliation (Kosut, 2006). Sociological accounts of late modernity read this shift as a means of anchoring identity against the transience of fashion, with the body serving as a durable canvas for the construction and display of the self (Sweetman, 1999). The consequence is a generationally uneven normative landscape: tattoos are at once a normalized marker of individuality for younger cohorts and a residual signal of unprofessionalism for institutions whose appearance codes were formed under the earlier deviance frame. Education is precisely such an institution, which makes the tattooed teacher a revealing site at which the older and the newer cultural readings of body art collide.

The tattooed educator offers a sharp test case, because institutional expectations, parental judgements, and student perceptions converge on appearance in a way that gives the accuracy of normative beliefs real professional consequences. Visible tattoos continue to carry residual workplace stigma despite growing societal acceptance, including in education (Timming, 2015; Broussard & Harton, 2017; Henle et al., 2022; Westerfield et al., 2012; Cerenado, 2024), and national evidence confirms the persistence of this stigma in Hungary (Karácsony et al., 2024). Physical education teachers are generally regarded as more acceptable when visibly tattooed than academic-subject teachers (Youngbauer, 2018). With tattoo prevalence among adults under 40 in Europe and North America estimated at 25–40% (Laumann & Derick, 2006), the normative positioning of tattooed educators is both timely and poorly understood. More broadly, visible appearance cues are known to generate trait inferences through halo effects and to shape educational judgements even when unrelated to competence (Dion et al., 1972; Langlois et al., 2000; Ritts et al., 1992; Bhowmik et al., 2025; McIntyre et al., 2020; Frühauf et al., 2024), supporting the premise that tattoos may function as socially meaningful cues in schools.

Pluralistic ignorance is the mechanism by which such collective misperception becomes institutionally entrenched: a majority privately rejects—or, conversely, privately accepts—a position while believing that most others hold the opposite view, generating public conformity to a consensus that has no genuine private endorsement (Prentice & Miller, 1993). The dynamic is self-reinforcing—each person conceals their private view believing themselves an outlier, so no corrective information circulates and the false norm persists. It has been documented across domains including college drinking (Prentice & Miller, 1993), organizational culture (Westphal & Bednar, 2005), and career choice (Leong et al., 2024), with its educational relevance now receiving systematic attention (Halbesleben et al., 2010; Barth & Grütter, 2025) and its formal conditions of emergence and stability increasingly well specified (Mendes et al., 2017; Lütz & Wardil, 2024; Gavrilets et al., 2026; Mata, 2025; Van Grootel et al., 2018).

Pluralistic ignorance is conceptually distinct from the broader study of how perceived norms shape attitudes. Research establishing that perceived norms drive outcomes independently of individuals’ own positions (Ward et al., 2025), and that anticipated social reactions produce real consequences even when inaccurate (Wang et al., 2025), treats perceived norms as exogenous inputs; it does not assess whether those norms are accurate. Pluralistic ignorance, by contrast, requires direct operationalization of a meta-perception gap—the comparison of what individuals privately hold with what they attribute to relevant reference groups. The existence, magnitude, and group-level variation in this gap is what the present study measures. Because perceived norms are formed inferentially from prior expectations and available social information (Dannals & Li, 2024) and can persist through feedback loops of public behaviour and institutional reinforcement even after private attitudes shift (Gelfand et al., 2024), they are especially vulnerable to error when attitudes are private and reference groups institutionally distant. Those with direct exposure to colleagues’ attitudes—such as in-service teachers—should therefore show smaller gaps than student groups, who must infer others’ positions from indirect cues (Miller & McFarland, 1991). The four-group design allows this gradient to be examined directly.

Beyond group membership, several individual factors should influence acceptance. Personal tattoo experience—having a tattoo, wanting one, or everyday contact with tattooed people—is among the most consistent predictors of positive tattoo attitudes, reflecting both self-selection and contact-based attitude change (Allport, 1954; Armstrong et al., 2004; Koch et al., 2010; Dickson et al., 2015). Gender effects are reported but inconsistent and complicated by gendered appearance expectations in occupational settings (Hawkes et al., 2004; Swami & Furnham, 2007; Baumann et al., 2016). For in-service teachers, length of professional experience may serve as a proxy for socialization into more conservative appearance norms: teachers who entered the profession when tattoos were more heavily stigmatized may hold more restrictive views, whereas younger teachers educated in a more permissive culture may be more accepting—consistent with generational evidence that younger cohorts report greater acceptance (Tews & Stafford, 2020; Weiler, 2024). Such individual orientations are increasingly recognized as substantive professional–psychological constructs rather than mere background (Teistler, 2024; Gómez Yepes et al., 2026).

The present research tested whether pluralistic ignorance operates in this domain. On the basis of the reviewed literature, we predicted that in all four groups own acceptance would exceed attributed acceptance for both teachers and parents (H1); that this gap would be smaller among in-service teachers than among student groups (H2); that PE-teacher acceptance would exceed academic-subject-teacher acceptance across groups, with no difference between PE-specialist and non-specialist teachers in their own acceptance (H3); that personal tattoo experience would positively predict acceptance (H4); and that, among in-service teachers, both age and years of experience would be negatively associated with acceptance (H5).

## 2. Materials and Methods

### 2.1. Participants and Procedure

Participants were recruited from four educational groups across multiple institutions in Hungary: PE teacher trainees (*n* = 92) and university students from non-PE disciplines (n = 606), both at the University of Debrecen; secondary school students (*n* = 155) from 32 Hungarian secondary schools; and in-service teachers (*n* = 373), of whom 79.9% reported PE as their main or combined teaching specialization (mean age 42.9 years, SD = 11.0; mean teaching experience 16.9 years, SD = 11.6). The total sample was *N* = 1226. Data were collected between December 2025 and February 2026 using an online questionnaire (Google Forms). Participation was voluntary and anonymous; participants were approached through institutional contacts and professional networks. All participants provided informed consent, and parental consent was obtained in advance for minor participants. The study was approved by the Ethics Committee of the Faculty of Economics and Business of the University of Debrecen (Approval No. GTK-KB 006/2026).

Recruitment proceeded through institutional and personal channels. PE teacher trainees and university students from non-PE disciplines were recruited through a Facebook group for students of the University of Debrecen, in which the questionnaire link was posted. Secondary school students were recruited indirectly: the link was sent to the PE teachers of 32 secondary schools in the Northern Great Plain and Northern Hungary regions, who were asked to have it completed by those of their students who were able to take part. In-service teachers were approached through the authors’ personal professional networks. No eligibility criteria were applied beyond current enrolment or employment in the relevant role at the time of data collection; the only exclusion criterion was withheld consent. Participant characteristics for each group are reported in Table 1.

### 2.2. Measures

*Instrument:* All constructs were assessed with a purpose-built questionnaire administered in Hungarian. Two parallel versions were used, identical in item content and wording except for the addressee: the student version referred to the respondent’s own teachers, the teacher version to the respondent’s colleagues. All items are reported in full in the Supplementary Material.

*Acceptance of Tattooed Teachers:* Participants rated their acceptance of a tattooed PE teacher and a tattooed academic-subject teacher separately (“How far would you accept it if, for example, the PE teacher had a tattoo that was also visible?”, and the same item naming the mathematics or Hungarian-language teacher), each on a five-point scale from 1 (not at all acceptable) to 5 (fully acceptable). These constituted the primary dependent variables (PE teacher acceptance; academic subject teacher acceptance). The two items were also treated as a two-item acceptance scale; internal consistency was high in every group (Cronbach’s α = 0.938, 0.901, 0.856 and 0.978 for PE trainees, university students, secondary school students and teachers respectively; α = 0.938 and Spearman–Brown coefficient = 0.943 in the total sample). The two items are nevertheless analyzed separately throughout, because H3 concerns the difference between them.

*Attributed Acceptance and Own Valence:* A single multiple-response item (“Does having a tattoo influence how a teacher is judged? Tick all that apply”) captured three valence judgements at once: the respondent’s own evaluation, the evaluation attributed to teachers in general, and the evaluation attributed to parents. For each of the three referents the respondent could tick a positive or a negative option; in the teacher version a neutral option was additionally offered for each referent. Respondents could leave any referent unticked. Own positive endorsement and attributed positive acceptance were coded as binary indicators from this item, and gap scores were computed as own positive minus attributed positive, separately for the teacher and the parent referent; positive values indicate that respondents attribute more disapproval to others than their own position warrants. Dichotomization follows established pluralistic-ignorance practice, which focuses on the directional structure of misperception rather than graded attitude intensity (Prentice & Miller, 1993; Van Grootel et al., 2018), while the Likert items provide the continuous measure of attitude strength. Because the response options were not identical across questionnaire versions, all gap analyses were repeated on a valence-conditional basis (see Section 2.3).

*Perceived Trendiness and Pattern Sensitivity:* Two further five-point items assessed the perception of tattoos as a social trend (“How fashionable do you think tattooing is nowadays?”) and the perceived influence of the tattoo’s design on judgements of the teacher (“Does the pattern of the tattoo—its form, for example a floral symbol, an abstract shape, a skull—influence how a teacher is judged?”; labelled pattern). Both items, and the two acceptance items above, used the same anchors: 1 (not at all) to 5 (completely).

*Personal Tattoo Experience:* A single four-option item (“Do you have a tattoo?”: no and do not want one/no but would like one/one/several) yielded two binary variables: tattoo status (one or several tattoos vs. none) and tattoo desire (no tattoo but would like one vs. all others).

*Tattoo Exposure:* A single four-option item asked whether the respondent knew any tattooed teachers (student version) or tattooed colleagues (teacher version). Responses were scored ordinally (0 = believes there are none, 1 = not aware of any, 2 = knows one, 3 = knows several) and entered as a single index in the regression models.

*Demographic and Professional Variables:* Participants reported gender, settlement type (coded ordinally from farmstead to capital), and—for in-service teachers—age and years of teaching experience. Subject specialization among teachers was coded as PE specialist versus other. Ten teachers gave years of experience in free-text form (e.g., “just started”, “two and a half”); these were recoded numerically before analysis.

*Single-Item Measures:* Several constructs—perceived trendiness, pattern sensitivity, tattoo status, tattoo desire and tattoo exposure—were assessed with single items, for which internal-consistency coefficients are not definable. Single-item operationalization was chosen because each construct is concrete, unidimensional and directly observable to the respondent, and because questionnaire length had to be kept short for the secondary school subsample. This is acknowledged as a limitation in Section 4.3.

### 2.3. Analytic Strategy

Group differences on Likert-scale variables were tested with Kruskal–Wallis tests and Bonferroni-adjusted Dunn post hoc comparisons, with η^2^ as the effect size. Within-group differences between PE and academic-subject teacher acceptance were tested with Wilcoxon signed-rank tests (effect size r). Group differences in dichotomous outcomes were tested with chi-square tests and Cramér’s V. Predictors of own positive attitude were modelled with binomial logistic regression (group membership, gender, tattoo status, tattoo desire, tattoo exposure, settlement type), with McFadden’s pseudo-R^2^ for fit. Among in-service teachers, associations between professional seniority and acceptance were examined with Spearman correlations, and PE-specialist versus non-specialist differences with Mann–Whitney U tests. To make fuller use of the multi-response items, exploratory binary indicators were derived to characterize the conditional structure of acceptance (unconditional vs. boundary-conditioned acceptance, categorical opposition, positive symbolic associations), the visibility of acceptable tattoo placements (grouped into arm, leg, torso, and highly visible zones), broader normative-attitudinal profiles, and the presence of teacher- and parent-referenced norm-misperception gaps; these are reported in condensed form below, with the full results in the Supplementary Material. Because the neutral response option for the valence item was offered only in the teacher version of the questionnaire, all gap analyses were additionally repeated on a valence-conditional basis, restricting each referent’s denominator to respondents who endorsed either the positive or the negative option. This places the four groups on a common metric and tests whether the group ordering of gap scores is an artefact of the differing option sets. All analyses were conducted in R (version 4.3.1).

Use of Generative AI: During the revision of this manuscript no generative AI assistant was used.

## 3. Results

### 3.1. Descriptive Statistics

Table 2 summarizes group-level descriptive statistics for the four primary outcome variables. All Likert-scale variables departed from normality, with acceptance medians at the scale ceiling, confirming the use of non-parametric tests throughout. Acceptance of tattooed teachers was high in all four groups on both items. The teacher group showed the most dispersed distributions, indicating greater internal heterogeneity than among the student groups, and secondary school students rated tattoo design quality (pattern) notably lower than all other groups.

### 3.2. Pluralistic Ignorance: Self-Reported Versus Attributed Acceptance

Table 3 presents self-reported and attributed acceptance with perception gap scores. H1 was supported in all four groups and for both reference targets: the proportion reporting personally positive attitudes substantially exceeded the proportion attributing positive attitudes to teachers or to parents. The gap was largest among secondary school students (gap_teachers = +0.697; gap_parents = +0.703) and university students (+0.620; +0.652), and much smaller among practising teachers (+0.118; +0.121). Group membership was strongly associated with self-reported positive attitude, χ^2^(3) = 351.469, *p* < 0.001, Cramér’s V = 0.535, and with attributed negativity toward teachers and parents, χ^2^(3) = 114.219, V = 0.305, and χ^2^(3) = 222.112, V = 0.426, respectively (both *p* < 0.001). H2 was supported: teacher gaps were the smallest of the four groups, yet remained positive—practising teachers still systematically overestimated the negativity of colleagues and parents despite far greater direct exposure to colleagues’ actual attitudes. Group membership was also significantly associated with perceived subject-dependency of acceptance, χ^2^(3) = 35.418, *p* < 0.001, V = 0.172, with PE teacher trainees most likely (38.0%) and secondary students least likely (12.3%) to believe acceptability depends on subject. The degree of this compression is, however, sensitive to the response format and is examined in Section Sensitivity Analysis: Valence-Conditional Gap Scores.

**Figure 1:**
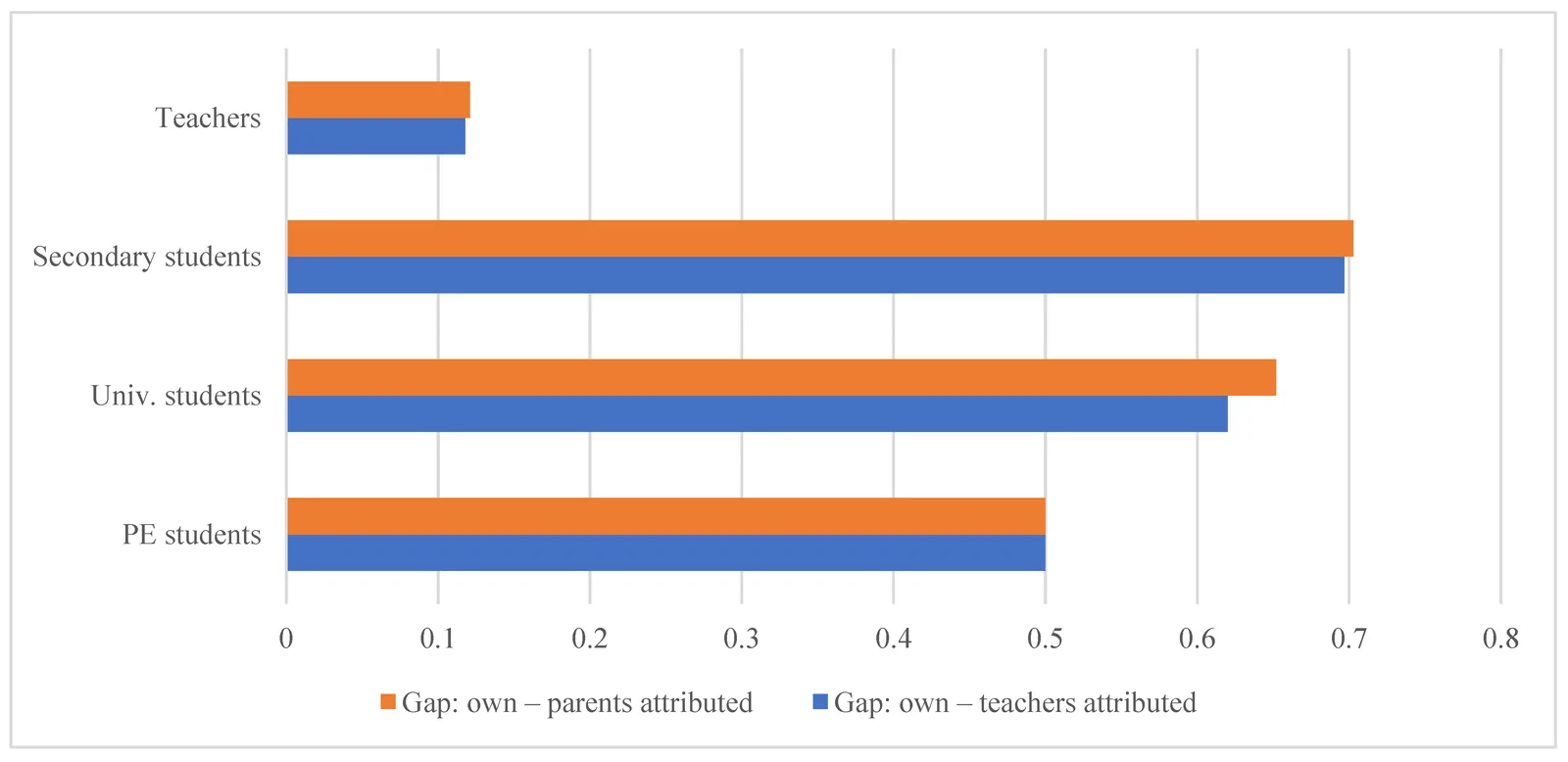
Perception gaps by group and reference target, shown as unconditional gap scores (cf. Table 4 for the valence-conditional values), expressed as the difference in the proportion endorsing a positive attitude (own minus attributed) for two reference groups—other teachers and parents—across the four educational groups.

The pattern of these gaps is summarized graphically in Figure 1, which makes visible the consistent positive discrepancy between own and attributed acceptance across all four groups and both reference targets, as well as its compression among in-service teachers.

#### Sensitivity Analysis: Valence-Conditional Gap Scores

Because the neutral option was available only in the teacher version, the gap scores in Table 3 are not strictly comparable across groups. All gap analyses were therefore repeated on a valence-conditional basis, restricting each referent’s denominator to respondents who endorsed either the positive or the negative option (Table 4). Two features of the primary analysis were preserved: the gap remained positive in all four groups and for both referents, and in-service teachers remained the group with the smallest gaps. The relative ordering of the three student groups, however, did not hold. Under the conditional specification the largest gaps were found among PE trainees and university students rather than among secondary school students, indicating that the rank order within the student groups is sensitive to the response format and should not be interpreted substantively. The teacher–student difference was also considerably reduced, from roughly one-sixth of the student gaps on the unconditional metric to approximately two-thirds on the conditional one. H1 is thus robust to the response-format difference, and H2 is supported in direction under both specifications, but the extent to which professional experience attenuates norm-misperception should be read as bounded by these two estimates rather than by the unconditional figure alone.

**Table 4:** Valence-conditional gap scores.

	PE Trainees	Univ. Students	Secondary Students	Teachers
Own positive, conditional (%)	86.0	94.4	94.7	57.1
Attributed: teachers positive, conditional (%)	5.0	14.0	21.8	10.2
Attributed: parents positive, conditional (%)	4.5	8.6	20.0	10.2
Gap: own − teachers attributed	+0.810	+0.804	+0.729	+0.469
Gap: own − parents attributed	+0.815	+0.858	+0.747	+0.469
Conditional n (own/teachers/parents)	57/60/67	462/428/474	132/78/80	98/118/108

Note. Denominator for each referent restricted to respondents endorsing either the positive or the negative option, so that groups are placed on a common metric irrespective of whether a neutral option was offered.

### 3.3. Group Differences in Acceptance Levels

Kruskal–Wallis tests revealed significant group differences on all four outcomes (Table 5). For PE teacher acceptance, university students scored highest (M = 4.779) and teachers lowest (M = 4.340). For academic subject teacher acceptance, university students again differed significantly from all other groups, which did not differ from one another after correction. The pattern variable showed the largest group differentiation (η^2^ = 0.052), driven by the markedly lower ratings of secondary school students.

### 3.4. PE Versus Academic Subject Teacher Acceptance

H3 was supported. Wilcoxon signed-rank tests confirmed that PE teacher acceptance exceeded academic subject teacher acceptance in all four groups, though absolute differences were small (0.053–0.271) and effect sizes modest (r = 0.161–0.390; Table 6). Within the teacher subsample, subject specialty did not predict acceptance. Teachers were classified as PE specialists if physical education was reported as their main or combined specialization (*n* = 298) and as non-specialists otherwise (*n* = 75), the same coding as in Section 2.1 and Table 1. Mann–Whitney comparisons were non-significant for PE teacher acceptance (U = 10,582, *p* = 0.387, r = 0.053), academic subject teacher acceptance (U = 10,810, *p* = 0.605, r = 0.033), and own positive attitude (U = 11,224, *p* = 0.926, r = 0.004). PE-specialist teachers were no more accepting of tattooed colleagues than non-specialists.

### 3.5. Predictors of Personal Positive Attitude

Binary logistic regression predicting own positive attitude fitted well (McFadden’s pseudo-R^2^ = 0.335; likelihood-ratio χ^2^(8) = 566.60, *p* < 0.001; Table 7). H4 was supported: all three tattoo-experience variables were significant positive predictors, with tattoo possession the strongest single indicator after group membership (OR = 8.19), followed by wanting a tattoo (OR = 5.05) and tattoo exposure (OR = 1.32 per unit). Group membership dominated overall: relative to teachers, secondary students were 41.4 times, university students 21.0 times, and PE trainees 10.3 times more likely to hold a positive attitude. Male gender predicted lower acceptance (OR = 0.624, *p* = 0.007); settlement type was non-significant (*p* = 0.768).

### 3.6. Professional Seniority Among In-Service Teachers

H5 was supported. Within the teacher subsample, both age and years of experience were negatively correlated with all three acceptance outcomes (Table 8). Correlations were modest but consistent; the association between years of experience and own positive attitude (ρ = −0.253) was numerically the largest, suggesting that cumulative professional socialization may predict conservative attitudes somewhat more strongly than chronological age alone, though the two variables are highly intercorrelated.

### 3.7. Conditional Acceptance, Visibility, and Norm-Misperception

The exploratory analyses qualify the core findings by showing that acceptance was largely conditional rather than absolute. Across groups, categorical rejection was uncommon, but so was fully unconditional acceptance; most respondents occupied a middle ground of conditional permissiveness, tolerating tattooed teachers while attaching limits to visibility, body location, and professional display. The most common restriction concerned the face, endorsed by roughly half of PE trainees, university students, and secondary students, and less often by teachers, who were instead more likely to reject tattoos from all teacher types or to state that teachers should not have tattoos. Younger groups thus tended to regulate tattoo visibility, whereas teachers more often invoked the professional role itself as the boundary. Positive symbolic associations showed the sharpest divide: secondary and university students were far more likely than teachers to describe tattooed teachers as cool or to attach positive traits to them (full boundary-setting indicators in Supplementary Table S1).

Acceptable tattoo placements followed a clear visibility gradient (Table 9; item-level detail in Supplementary Table S2). Arm placements were most widely accepted, followed by leg and torso placements, while highly visible placements—hand, neck, nape, and face—were least accepted. Forearm tattoos were accepted by more than half of the three student groups but by only a quarter of teachers, who showed the most restrictive pattern overall and were most likely to select no acceptable placement. This indicates that the teacher group’s lower acceptance is specifically organized around professional visibility and display rather than reflecting a uniformly negative attitude, and that body location may be a more powerful organizing principle than teacher subject type.

Five non-exclusive normative-attitudinal profiles were derived (Table 10). Conditional professional acceptance was the most common profile in most groups, especially among PE trainees and university students. Enthusiastic normalization was strongly group-dependent (present in 59.7% of university and 65.8% of secondary students but only 24.7% of teachers), whereas traditional restriction was most common among teachers (21.7%). The general norm-misperceiver profile—personally positive but attributing non-acceptance to both teachers and parents—was frequent among students (up to 65.2%) but rare among teachers (12.9%; Cramér’s V = 0.442), confirming that norm-misperception is strongly structured by institutional position.

Two logistic models predicting teacher- and parent-referenced misperception gaps (Supplementary Tables S3 and S4) confirmed this structure. Norm-misperception was driven primarily by group position—all student groups, and especially secondary students, had substantially higher odds of carrying a gap than teachers—and by personal tattoo experience and perceived trendiness. Notably, contact with tattooed teachers or colleagues did not independently predict gap membership once group and other variables were controlled, although it retained a small positive effect on Likert-scale acceptance in adjusted models (PE teacher acceptance B = 0.101, *p* = 0.027; academic subject teacher acceptance B = 0.117, *p* = 0.027). Direct contact may therefore soften personal evaluations without correcting beliefs about the wider normative climate. The face-related restriction predicted the parent-referenced gap but not the teacher-referenced gap, suggesting that highly visible tattoos activate assumptions about parental scrutiny more strongly than assumptions about peer judgement.

## 4. Discussion

The present research examined whether individuals privately accept tattooed teachers more than they attribute acceptance to relevant reference groups, and whether this meta-perception gap varies across stakeholder groups. Across all four educational groups in Hungary, private acceptance substantially exceeded attributed acceptance, providing evidence consistent with pluralistic ignorance as a systemic feature of the normative climate surrounding tattooed teachers.

### 4.1. A Collective Misperception: What the Gap Reveals

The most striking feature of the data is not the existence of a self–other gap—expected on theoretical grounds—but its scale and the group that anchors it. In-service teachers, the group with the greatest direct access to colleagues’ genuine attitudes, still showed a positive gap between their own acceptance and the acceptance they attributed to other teachers and parents, albeit a substantially compressed one. This suggests that professional appearance norms in education are sustained at least partly by self-reinforcing misperception of the normative climate rather than by genuine attitudinal consensus. The finding aligns with evidence that perceived norms function as independent drivers of attitudes over and above private positions (Ward et al., 2025); what the present data add is a systematic self–other discrepancy in attributed norms—the consensus individuals believe they are conforming to is considerably more conservative than their own private attitudes. This distinction between the effects of perceived norms and the accuracy of norm perception is the specific contribution of the pluralistic-ignorance framework, and it helps explain why conservative appearance norms persist even as cohort attitudes shift (Dannals & Li, 2024; Gelfand et al., 2024).

The persistence of a positive gap even among teachers is especially informative. Teachers who interact with colleagues daily, and who may themselves have tattooed colleagues, still attributed substantially less acceptance to others than they reported themselves—consistent with evidence that pluralistic ignorance can survive in groups with high internal transparency when the topic is socially sensitive and public expression is suppressed by anticipated disapproval (Westphal & Bednar, 2005). Norm-enforcing dynamics that require no deliberate coordination (Konieczna, 2024) may operate here: teachers who privately accept tattooed colleagues refrain from voicing this acceptance, denying others the corrective information that would reduce misperception. Professional culture may thus function not as a passive backdrop but as an active mechanism sustaining a perceived conservative norm.

The contrast between teachers and student groups—especially secondary students, who showed the largest gaps—reveals a developmental and institutional gradient. Secondary students attributed overwhelming negativity to teachers and parents while themselves holding largely positive attitudes, a pattern consistent with findings that anticipated exclusion based on assumed group beliefs produces real attitudinal consequences even when those beliefs are inaccurate (Wang et al., 2025; Miller & McFarland, 1991), and that such misperception may discourage adolescents from expressing counter-normative appearance attitudes (Kilmartin et al., 2008).

The regression results add texture. Group membership dominated, but the tattoo-experience variables each contributed independently: tattoo possession increased the odds of a positive attitude more than eightfold, consistent with a contact-based account in which direct, bodily involvement in tattoo culture is linked to more positive evaluations of tattooed others even in professional settings (Allport, 1954; Timming, 2015; Karácsony et al., 2024). As tattoo prevalence rises in younger cohorts, this pattern may signal a slow drift toward greater acceptance that could eventually erode the current conservative norm. The modest but robust gender effect—with men being less accepting—runs counter to the general tattoo-attitudes literature (Hawkes et al., 2004; Swami & Furnham, 2007; Baumann et al., 2016). Since the present design assessed neither adherence to traditional professional norms nor gender-specific appearance expectations, the finding is reported here as an observation calling for targeted replication rather than as a basis for interpretation.

The null finding for subject specialty within the teacher group is theoretically informative. Although respondents across all groups rated tattooed PE teachers as more acceptable than tattooed academic-subject teachers, PE-specialist teachers were no more accepting of tattooed colleagues than non-specialists. The PE-superiority effect thus appears to operate largely in the minds of outside observers rather than as a genuine attitudinal distinction within the profession (Youngbauer, 2018). This comparison, however, rests on a markedly unbalanced subsample: 79.9% of in-service teachers reported PE as their main or combined specialization, leaving a substantially smaller non-PE comparison group. The null result is therefore best read as the absence of a large specialty difference rather than as evidence of equivalence, and the teacher findings as a whole characterize a predominantly PE-specialist teaching population rather than the Hungarian teaching profession at large. Finally, the negative associations of age and experience with acceptance, with experience being the slightly stronger correlate, are consistent with a professional-socialization effect operating over and above generational replacement (Ward et al., 2025; Tews & Stafford, 2020): longer-serving teachers may have more fully absorbed conservative appearance norms, so that the injunctive norm functions as a stable anchor even where private attitudes are more liberal.

### 4.2. Conditional Acceptance and the Professional Visibility Boundary

The exploratory analyses qualify the main finding in an important way: the central question is not whether tattooed teachers are accepted, but under what visual and professional conditions. Most respondents were neither categorical rejecters nor unconditional acceptors but conditional permissives, negotiating a boundary between personal expression and professional role performance. A tattoo was acceptable on the arm, leg, or a coverable area but less so on the face, neck, or hand—a visibility hierarchy indicating that the professional meaning of tattoos is not intrinsic but depends on location, public visibility, and perceived interference with the symbolic expectations of the teacher role.

The face-related restriction is especially revealing: even relatively permissive groups maintained a boundary around forms of body modification that are hard to conceal and strongly tied to identity display. Among teachers this specific restriction was less common, but teachers were more likely to invoke the professional role itself as the boundary. The placement data reinforce this: teachers showed the most restrictive pattern, particularly for visible areas, suggesting they evaluate tattoos through a display-management framework in which the issue is whether the tattoo enters the professional field of visibility. Body location may thus be a more powerful organizing principle than teacher subject type, making professional appearance norms multidimensional—involving the teacher’s subject, the tattoo’s location and visibility, and the audience before whom it is displayed.

The normative profiles show that acceptance is heterogeneous: unconditional acceptors, enthusiastic normalizers, conditional professionals, and traditional restrictors coexist. This heterogeneity helps explain why pluralistic ignorance can persist even in a generally accepting sample: when the majority is conditionally rather than uniformly permissive, the true normative climate is hard to infer, public silence is misread as disapproval, and conditional acceptance is mistaken for rejection. The gap models sharpen this: norm-misperception was concentrated among those for whom tattoo acceptance is personally or culturally salient—students, the tattooed or tattoo-interested, and those who view tattoos as culturally current—but who perceive the educational institution as more conservative than themselves. This points to a perceived temporal mismatch in which tattoos are seen as normalized in wider society while schools and parents are imagined to lag behind.

Contact had a more limited role than personal tattoo experience: it improved respondents’ own evaluations but did not independently correct beliefs about others’ attitudes. Pluralistic ignorance is therefore not simply a deficit of exposure to tattooed individuals but a deficit of reliable information about the distribution of others’ private attitudes, which is why even direct contact may fail to correct it unless aggregate peer attitudes are made explicitly visible. The implication for practice is that contact-based initiatives shift individual attitudes only modestly and cannot substitute for deliberate norm-correction that communicates actual peer acceptance—an approach shown elsewhere to promote inclusion and change behaviour by signalling peers’ attitudes and working through socially influential actors (Tankard & Paluck, 2016; Murrar et al., 2020; Paluck et al., 2016). The parent-reference findings add nuance: parents, as an external evaluative public imagined as moral stakeholders, may function as an anticipated source of reputational risk—especially for highly visible tattoos—even when their actual attitudes are unknown.

These boundary conditions are best understood against the trajectory of tattooing as a cultural practice. The visibility hierarchy observed here—arms acceptable, face and neck not—maps closely onto the historical fault line between tattooing as a concealable personal body project and tattooing as an irreversible public declaration of identity (Sweetman, 1999). As tattooing has moved from a marker of subcultural affiliation to an artified and commodified mainstream practice (Kosut, 2006, 2014), the terms of institutional resistance appear to have shifted rather than dissolved: what is contested is no longer whether a teacher may be tattooed, but how far the tattoo may be seen. This suggests a plausible trajectory. If the normalization of body art continues along its present course, and if rising prevalence among younger cohorts feeds into the teaching profession, the visibility threshold may migrate outward over time rather than disappear—the professional boundary relocating to progressively more visible zones while remaining, in principle, intact. Whether the threshold moves, and how fast, is an empirical question that repeated cross-sectional measurement in this domain could answer (Kosut, 2008; Lane, 2014).

### 4.3. Limitations and Future Directions

Several caveats qualify these findings. First, the cross-sectional design precludes causal inference: the inverse relationship between experience and acceptance is consistent with socialization but could also reflect a cohort effect, since more senior teachers came of age when tattoos were more stigmatized. Longitudinal or cohort-sequential designs are needed to disentangle these accounts. Second, the sample, though large, was drawn from a single national context; the social meaning and professional consequences of body art vary across cultures (Adisa et al., 2021), and settings where tattoos are more prevalent and professional standards less rigid might show higher baseline acceptance and different gap patterns, so cross-national replication remains a desideratum. Third, the meta-perception gap was measured indirectly: participants estimated others’ reactions, but no independent measure of actual peer attitudes was collected within the same sample. Strictly, the study documents a systematic self–other discrepancy in attributed attitudes that is consistent with—but cannot conclusively establish—pluralistic ignorance. Several features nonetheless favour a misperception interpretation: the gap was large and consistent across all four groups and both reference targets, varied across groups exactly as differential access to peer attitudes would predict, and was structured by theoretically expected predictors—patterns that a uniform positivity bias would not produce, and that match the proxy logic of group-level pluralistic-ignorance measurement (Prentice & Miller, 1993; Van Grootel et al., 2018). A stronger design would collect both self- and peer-report within the same sample and test whether corrective information about peer attitudes reduces the gap. Fourth, the questionnaire versions were not fully equivalent. A neutral response option for the valence item was offered to in-service teachers but not to the three student groups, and the teacher group made heavy use of it (63.5% for their own evaluation). The unconditional gap scores for teachers are therefore not directly comparable with those of the student groups. The sensitivity analysis reported in Section Sensitivity Analysis: Valence-Conditional Gap Scores addresses this by placing all groups on a valence-conditional metric, under which the positive direction of the gaps and the position of teachers as the group with the smallest gaps are preserved, though the ordering of the three student groups is not, and the teacher–student difference is substantially smaller. Future work in this area should administer an identical response format to all groups. A related constraint is that several predictors—perceived trendiness, pattern sensitivity, tattoo status, tattoo desire and tattoo exposure—were assessed with single items, which precludes estimation of their internal consistency and may attenuate observed associations. A further constraint is that participant age was recorded only for in-service teachers, so the four groups cannot be compared on age directly, and the teacher subsample was heavily weighted toward PE specialists (79.9%), which limits generalization to the wider teaching profession. Fifth, the study addressed only the evaluative dimension and not behavioural consequences; whether this misperception translates into self-censorship, concealment, or anticipatory decisions against obtaining tattoos among prospective teachers is a question of direct practical relevance that the present data cannot answer (Frühauf et al., 2024; Lerner & Lissitsa, 2025). Qualitative and mixed-methods approaches would be especially valuable here. Finally, recruitment was non-probabilistic throughout. University participants were reached through a single institution’s student social-media group, and secondary school students were recruited via their PE teachers, who selected which of their students were invited. The student samples are therefore self-selected and, in the secondary school case, mediated by a gatekeeper whose own subject affiliation may be related to the attitudes under study.

## 5. Conclusions

Across four educational groups in Hungary, private acceptance of tattooed teachers consistently and substantially exceeded attributed acceptance, for both teacher and parent reference groups, and even among in-service teachers with direct access to colleagues’ genuine attitudes. The apparent norm of disapproval in Hungarian educational settings thus appears to rest on a collectively sustained self–other discrepancy rather than on genuine consensus. Theoretically, the study contributes to the literature on norm misperception in education by directly operationalizing the meta-perception gap rather than examining the effects of perceived norms, and shows that gap to be both large and structurally patterned; the persistence of a positive gap among teachers suggests that professional culture actively maintains the perceived norm through the public suppression of private acceptance. Practically, the findings suggest that simple disclosure interventions—making actual peer attitudes visible within school communities—could reduce conformity pressure and create space for more authentic professional self-expression, with the largest potential impact among secondary students (whose gaps were largest) and senior teachers (whose socialization is consistent with deepened misperception). The additional analyses show that this norm is sustained not only through general disapproval but through conditionality: acceptance was highest for coverable placements and lowest for highly visible ones such as the face, neck, or hand. Norm correction therefore requires more than demonstrating general acceptance; it requires making explicit which appearance expectations are genuinely shared, which are merely assumed, and which persist because individuals continue to mistake silence for consensus. Ultimately, tattooed teachers in Hungary appear to be navigating a norm that is considerably less widely shared than it seems—one sustained not by genuine consensus but by the systematic underestimation of others’ acceptance.

## Figures and Tables

**Table 1:** Participant characteristics by group.

Characteristic	PE Trainees (*n* = 92)	Univ. Students (*n* = 606)	Secondary Students (*n* = 155)	Teachers (*n* = 373)
Female (%)	35.9	82.7	71.0	71.3
Male (%)	64.1	17.3	29.0	28.7
Age, M (SD)	not collected	not collected	not collected	42.9 (11.0)
Has at least one tattoo (%)	28.3	34.5	12.9	38.9
one tattoo (%)	13.0	11.6	4.5	13.4
several tattoos (%)	15.2	22.9	8.4	25.5
No tattoo, would like one (%)	43.5	39.8	63.2	10.5
No tattoo, does not want one (%)	28.3	25.7	23.9	50.7
Exposure: knows several tattooed teachers/colleagues (%)	17.4	21.3	25.8	54.7
Exposure: knows one (%)	31.5	16.2	14.8	18.2
Exposure: not aware of any (%)	34.8	48.7	44.5	22.0
Exposure: believes there are none (%)	16.3	13.9	14.8	5.1
Settlement: village, small municipality or farmstead (%)	18.4	19.8	36.8	16.1
Settlement: town (%)	81.5	77.4	62.6	68.1
Settlement: capital (%)	0.0	2.8	0.6	15.8
Teaching experience in years, M (SD)	-	-	-	16.9 (11.6)
PE as main or combined specialization (%)	-	-	-	79.9

Note: Age was recorded only in the teacher version of the questionnaire and is therefore unavailable for the three student groups. Tattoo status and tattoo desire were derived from a single four-option item. Exposure refers to awareness of tattooed teachers (student groups) or tattooed colleagues (teachers). Percentages may not sum to 100 owing to rounding.

**Table 2:** Descriptive statistics for primary outcome variables by group (M (SD)/Mdn [IQR]).

Variable	PE Students (*n* = 92)	Univ. Students (*n* = 606)	Secondary Students (*n* = 155)	Teachers (*n* = 373)
PE teacher acceptance	4.554 (0.830)/5.0 [4–5]	4.779 (0.643)/5.0 [5–5]	4.677 (0.738)/5.0 [5–5]	4.340 (1.136)/5.0 [4–5]
Academic subject teacher acceptance	4.402 (0.995)/5.0 [4–5]	4.660 (0.839)/5.0 [5–5]	4.406 (1.011)/5.0 [4–5]	4.287 (1.148)/5.0 [4–5]
Perceived trendiness	4.185 (0.889)/4.0 [4–5]	4.221 (0.838)/4.0 [4–5]	4.135 (0.838)/4.0 [4–5]	3.877 (1.103)/4.0 [3–5]
Pattern	3.967 (1.104)/4.0 [3–5]	3.919 (1.146)/4.0 [3–5]	2.974 (1.409)/3.0 [2–4]	3.670 (1.208)/4.0 [3–5]

Note. Acceptance items 1–5; perceived trendiness = perceived trendiness of tattooing (1–5); pattern = perceived influence of the tattoo’s design on judgements of the teacher (1–5). IQR = interquartile range.

**Table 3:** Self-reported and attributed acceptance of tattooed teachers by group, with perception gap scores.

	PE Trainees	Univ. Students	Secondary Students	Teachers
Own positive (%)	53.3	71.9	80.6	15.0
Own neutral (%)	n/a	n/a	n/a	63.5
Own negative (%)	8.7	5.0	5.2	11.5
Own, no option endorsed (%)	38.0	23.1	14.2	10.0
Attributed: teachers positive (%)	3.3	9.9	11.0	3.2
Attributed: teachers neutral (%)	n/a	n/a	n/a	45.8
Attributed: teachers negative (%)	62.0	62.2	39.4	29.0
Attributed: teachers, no option endorsed (%)	34.7	27.9	49.6	22.0
Attributed: parents positive (%)	3.3	6.8	10.3	2.9
Attributed: parents neutral (%)	n/a	n/a	n/a	45.0
Attributed: parents negative (%)	69.6	73.6	42.6	26.8
Attributed: parents, no option endorsed (%)	27.1	19.6	47.1	25.3
Gap: own − teachers attributed	+0.500	+0.620	+0.697	+0.118
Gap: own − parents attributed	+0.500	+0.652	+0.703	+0.121

Note. All valence judgements derive from a single multiple-response item on which respondents could tick any combination of options, or none; percentages therefore do not sum to 100. A neutral option was offered for each referent in the teacher version of the questionnaire only, and is marked n/a for the three student groups. “No option endorsed” denotes respondents who ticked neither the positive nor the negative (nor, where available, the neutral) option for that referent. Gap scores = proportion endorsing own positive minus proportion attributing a positive reaction to the reference group.

**Table 5:** Kruskal–Wallis tests and Dunn post hoc comparisons for primary outcome variables.

Variable	H(3)	*p*	η^2^	Significant Pairwise Contrasts (Bonferroni)
PE teacher acceptance	52.324	<0.001	0.040	Univ. > Teachers (*p* < 0.001); Secondary > Teachers (*p* = 0.014); Univ. > PE students (*p* = 0.005)
Academic subject teacher acceptance	39.381	<0.001	0.030	Univ. > Secondary (*p* = 0.004); Univ. > Teachers (*p* < 0.001); Univ. > PE students (*p* = 0.012)
Perceived trendiness	22.020	<0.001	0.016	Univ. > Teachers (*p* < 0.001); all other contrasts n.s.
Pattern	66.635	<0.001	0.052	Secondary < all other groups (all *p* < 0.001); Univ. > Teachers (*p* = 0.005)

Note. N = 1226. η^2^ = eta-squared. n.s. = not significant after Bonferroni correction.

**Table 6:** Wilcoxon signed-rank tests comparing acceptance of tattooed PE versus academic subject teachers, by group.

Group	M PE Teacher	M Academic Subj.	W	*p*	R
PE students	4.554	4.402	6	0.002	0.325
Univ. students	4.779	4.660	20	<0.001	0.263
Secondary students	4.677	4.406	0	<0.001	0.390
Teachers	4.340	4.287	55	0.002	0.161

Note. r = rank-biserial correlation as effect size.

**Table 7:** Binary logistic regression predicting personally positive attitude toward tattooed teachers.

Predictor	B	SE	OR	95% CI	*p*
Intercept	−3.740	0.495	0.024	[0.009, 0.063]	<0.001
Univ. students (vs. teachers)	3.045	0.222	21.015	[13.595, 32.485]	<0.001
Secondary students (vs. teachers)	3.724	0.310	41.430	[22.572, 76.046]	<0.001
PE students (vs. teachers)	2.334	0.310	10.318	[5.616, 18.956]	<0.001
Male gender	−0.472	0.174	0.624	[0.444, 0.877]	0.007
Has a tattoo	2.103	0.201	8.187	[5.526, 12.129]	<0.001
Would like a tattoo	1.620	0.183	5.052	[3.527, 7.235]	<0.001
Tattoo exposure index	0.276	0.082	1.318	[1.122, 1.548]	0.001
Settlement type	0.030	0.101	1.030	[0.846, 1.254]	0.768

Note. N = 1226. Reference category for group = teachers. OR = odds ratio. McFadden’s pseudo-R^2^ = 0.335.

**Table 8:** Spearman rank correlations between age, years of experience, and acceptance variables within the teacher subsample.

	PE Teacher Acceptance	Academic Subject Teacher Acceptance	Own Positive Attitude
Age	ρ = −0.246 ***	ρ = −0.230 ***	ρ = −0.209 ***
Years of experience	ρ = −0.196 ***	ρ = −0.187 ***	ρ = −0.253 ***

Note. n = 367–372. *** *p* < 0.001.

**Table 9:** Acceptance of tattoo-placement zones by group.

Placement Zone/Response	PE Students	Univ. Students	Secondary Students	Teachers	χ^2^	*p*	V
Arm zone	69.6%	64.7%	66.5%	39.4%	73.35	<0.001	0.245
Leg zone	51.1%	48.5%	39.4%	33.2%	25.31	<0.001	0.144
Torso zone	53.3%	43.2%	24.5%	36.7%	26.54	<0.001	0.147
Highly visible (hand/neck/nape/face)	21.7%	21.1%	28.4%	9.1%	35.34	<0.001	0.170
Any placement	10.9%	15.7%	16.1%	8.3%	12.67	0.005	0.102
No placement	6.5%	5.8%	3.9%	18.5%	51.76	<0.001	0.205

Note. V = Cramér’s V.

**Table 10:** Normative-attitudinal profile indicators by group.

Profile Indicator	PE Students	Univ. Students	Secondary Students	Teachers	*p*	V
Unconditional acceptor	17.4%	27.6%	25.8%	30.8%	0.072	0.076
Conditional professionalist	73.9%	61.4%	54.8%	52.0%	<0.001	0.123
Enthusiastic normalizer	47.8%	59.7%	65.8%	24.7%	<0.001	0.331
Traditional restrictor	13.0%	8.6%	9.0%	21.7%	<0.001	0.175
General norm-misperceiver	48.9%	60.6%	65.2%	12.9%	<0.001	0.442

Note. Profile indicators are theoretically derived and not mutually exclusive. General norm-misperceiver: own positive = 1 AND attributed teacher positive = 0 AND attributed parent positive = 0. V = Cramér’s V.

## Data Availability

The original contributions presented in this study are included in the article and its Supplementary Material. Further inquiries can be directed to the corresponding author.

## Supplementary Materials

The following supporting information can be downloaded at: https://www.mdpi.com/article/10.3390/ejihpe16080115/s1. Table S1: Conditional acceptance and professional boundary-setting by group; Table S2: Acceptable tattoo placements by group (item-level); Table S3: Logistic regression predicting teacher-referenced norm–misperception gap; Table S4: Logistic regression predicting parent-referenced norm–misperception gap.
