# Supplementary material for: The Illusion of Disapproval: Pluralistic Ignorance and Attitudes Toward Tattooed Teachers
Source: Eur J Investig Health Psychol Educ. 2026 Aug 11;16(8):115. doi: 10.3390/ejihpe16080115 (PMC13512391; doi:10.3390/ejihpe16080115)
Supplement: Supplementary file 1 [file ejihpe-16-00115-s001.zip › ejihpe-4428724-supplementary.pdf]

Supplementary Materials

The following tables present the full results of the exploratory analyses summarized in Section 3.7. They are intended for submission as online Supplementary Materials rather than in the main text.

Table S1. Conditional acceptance and professional boundary-setting by group.

Indicator	PE st.	Univ. st.	Sec. st.	Teachers	χ^2	p	V
Unconditional / professional-irrelevance acceptance	88.0%	88.9%	78.1%	76.4%	31.61	< .001	.161
"Yes, but not on the face"	48.9%	47.2%	46.5%	30.0%	31.73	< .001	.161
"Acceptable, but should be covered"	18.5%	7.1%	5.8%	16.4%	30.66	< .001	.158
"A teacher should not have tattoos"	6.5%	2.8%	2.6%	9.9%	26.29	< .001	.146
"It is cool if they have one"	19.6%	30.7%	40.0%	9.4%	79.87	< .001	.255
Tattooed appearance does not influence evaluation	71.7%	79.4%	67.7%	75.9%	10.53	.015	.093
Positive traits associated with tattooed teachers	44.6%	54.1%	58.7%	22.0%	112.08	< .001	.302
Would accept tattoos from all teacher types	75.0%	86.3%	67.7%	75.1%	36.22	< .001	.172
Would accept tattoos from no teacher type	5.4%	3.0%	3.9%	16.1%	62.82	< .001	.226

Note. V = Cramér's V.

Table S2. Acceptable tattoo placements by group (item-level).

Tattoo placement	PE students	Univ. students	Secondary students	Teachers	p	V
Forearm	54.3%	54.0%	55.5%	24.9%	< .001	.271
Upper arm	53.3%	52.1%	41.9%	33.2%	< .001	.172
Calf	41.3%	38.4%	27.7%	22.5%	< .001	.160
Shoulder blade	29.3%	30.7%	9.0%	29.0%	< .001	.157
Lower leg	29.3%	31.4%	16.1%	16.9%	< .001	.166
Chest	30.4%	27.7%	9.7%	15.0%	< .001	.181
Thigh	25.0%	24.1%	9.7%	12.3%	< .001	.162
Spine/back	21.7%	23.3%	8.4%	11.3%	< .001	.166
Hand	15.2%	14.9%	21.9%	5.9%	< .001	.156
Neck	6.5%	8.1%	11.0%	2.4%	< .001	.120
Nape	7.6%	9.4%	7.1%	3.5%	.007	.100

Note. V = Cramér's V.

Table S3. Logistic regression predicting teacher-referenced norm–misperception gap.

Predictor	OR	95% CI	p
PE students vs. teachers	8.20	4.53–14.86	< .001
University students vs. teachers	12.22	8.08–18.49	< .001
Secondary students vs. teachers	19.50	11.22–33.90	< .001
Male gender	0.69	0.50–0.97	.031
Has a tattoo	4.84	3.28–7.15	< .001
Would like a tattoo	3.26	2.29–4.65	< .001
Knows a tattooed teacher/colleague	1.20	0.89–1.62	.238
Perceived trendiness	1.63	1.35–1.96	< .001
Pattern importance	0.93	0.82–1.05	.256
Perceived subject-dependency	0.98	0.71–1.36	.899

Predictor	OR	95% CI	p
Face-related restriction	1.28	0.96–1.70	.087

Note. McFadden's pseudo-R^2 = .287; likelihood-ratio χ^2 = 486.90, $p < .001$. Reference category for group = practising teachers.

Table S4. Logistic regression predicting parent-referenced norm–misperception gap.

Predictor	OR	95% CI	p
PE students vs. teachers	6.92	3.86–12.40	< .001
University students vs. teachers	12.26	8.17–18.41	< .001
Secondary students vs. teachers	16.75	9.70–28.91	< .001
Male gender	0.76	0.55–1.06	.107
Has a tattoo	4.28	2.91–6.29	< .001
Would like a tattoo	3.54	2.49–5.04	< .001
Knows a tattooed teacher/colleague	1.22	0.90–1.66	.196
Perceived trendiness	1.54	1.28–1.85	< .001
Pattern importance	0.89	0.78–1.00	.056
Perceived subject-dependency	0.95	0.68–1.31	.738
Face-related restriction	1.45	1.09–1.92	.011

Note. McFadden's pseudo-R^2 = .288; likelihood-ratio χ^2 = 488.77, $p < .001$. Reference category for group = practising teachers.
